# Evolution of Our Understanding of Myeloid Regulatory Cells: From MDSCs to Mregs

**DOI:** 10.3389/fimmu.2014.00303

**Published:** 2014-07-02

**Authors:** Masoud H. Manjili, Xiang-Yang Wang, Scott Abrams

**Affiliations:** ^1^Department of Microbiology and Immunology, Virginia Commonwealth University, Richmond, VA, USA; ^2^Massey Cancer Center, Virginia Commonwealth University, Richmond, VA, USA; ^3^Department of Human and Molecular Genetics, Virginia Commonwealth University, Richmond, VA, USA; ^4^Roswell Park Cancer Institute, Buffalo, NY, USA

**Keywords:** myeloid-derived suppressor cells, myeloid regulatory cells, *Nippostrongylus*, *Leishmania*, helminths, immunotherapy of cancer

## Abstract

The term myeloid-derived suppressor cells (MDSCs) was first suggested in 2007 in order to reflect the origin and function of myeloid cells during immunosuppression in cancer and other pathologic conditions. Emerging evidence suggests that MDSCs suppress CTL and Th1 responses in malignant diseases while they regulate effective immune responses in parasitic and helminth infections as well as Th17 inflammatory response during autoimmune diseases. Based on these data, the term myeloid regulatory cells (Mregs) more accurately reflects their function and interactions with different cells of the immune system during diseased conditions. Here, we provide evidence on the multifaceted function of Mregs during diseased states.

## Mregs Operate as MDSCs for the Suppression of Anti-Tumor Immune Responses

Myeloid-derived suppressor cells (MDSCs) were originally described as immunosuppressive cells of myeloid origin. These cells are known to be a heterogeneous mixture of myeloid cells at different stages of differentiation. They were initially named “immature myeloid cells” or “myeloid suppressor cells” (MSC) until they matured into MDSCs in 2007 (1). Most of the attention was initially focused on the role of these cells in cancer, because tumor-derived factors were shown to facilitate the production of MDSCs as well as their accumulation in the secondary lymphoid tissues and at the tumor site. In mice, MDSCs are broadly defined by the expression of CD11b and Gr-1, and could be subdivided into monocytic (CD11b^+^Ly6C^hi^Ly6G^−^) and granulocytic (CD11b^+^Ly6C^low^Ly6G^+^) subsets (2). In human being, MDSCs are characterized by the expression of CD33^+^CD11b^+^HLA-DR^−/low^. Human MDSCs are classified into monocytic and granulocytic subsets based on Lin^−^CD11b^+^CD14^+^CD15^−^and Lin^−^CD11b^+^CD14^−^CD15^+^, respectively (3). In fact, MDSCs reflect a mechanism by which myeloid regulatory cells (Mregs) suppress the host’s anti-tumor immune responses in favor of the tumor.

The ability of MDSCs to suppress anti-tumor Th1 and CTL immune responses has been demonstrated by their direct and indirect impacts on the immune system. The direct immune suppressive function of MDSCs is accomplished through at least three different pathways which include contact-dependent and/or contact-independent suppression of effector T cells (4–8), induction of Tregs (9), and inhibition of T cell trafficking (10). Contact-dependent suppression of T cells by MDSCs causes nitration of tyrosine residues in the TcR–CD8 complex, thereby disrupting the tumor antigen-MHC class I recognition by the TcR (4). Suppression of T cell proliferation during stimulation with anti-CD3/CD28 antibodies was also shown to be due to a contact-dependent mechanism (5). Cell contact is also required for MDSC-mediated suppression of NK cells in patients with hepatocellular carcinoma (6). It was reported that membrane-bound TGF-β1 on MDSCs is responsible for MDSC-mediated suppression of NK cell cytotoxicity, NKG2D expression, and IFN-γ production (11). Recently, it was reported that granulocytic MDSC subset can negatively regulate NK cell activation and function in response to vaccinia virus infection via producing reactive oxygen species (12). Most groups have found contact-independent mechanisms of T cell suppression by MDSCs releasing soluble factors such as IDO, arginase-1, nitric oxide, reactive oxygen species, and peroxynitrites (13, 14). MDSCs also produce IL-10 and TGF-β, resulting in the induction of Tregs in an antigen-specific manner (15). Finally, MDSCs can downregulate the expression of CD62L, which is an important receptor for T cell homing to the lymph nodes. This is accomplished by the expression of a disintegrin and metalloproteinase (ADAM)-17 on MDSCs which cleaves and results in shedding of the ectodomain of CD62L (10).

Indirect mechanisms of T cell suppression by MDSCs, which are mediated by granulocytic subset, include expression of matrix metalloproteinases (MMPs). MMPs can support the bioavailability of VEGF, thereby acting as tumor angiogenic factors; MMPs can also help to break down the extracellular matrix, facilitating dissemination and metastasis of the tumor (16).

## Mregs Modulate Th1 Response and Support Th2 Response: Protective Immune Responses Against Parasitic and Helminth Infections

While Mregs are harmful to anti-tumor immunity and some other diseases in which a robust Th1 response or CTL response is required, the ability of the Mregs to limit and modulate Th1 responses or support a skewed Th2 immunity could be beneficial during parasitic and helminth infections. The Th1 modulatory function of Mregs is evident during infection with intracellular protozoan parasites such as *Leishmania* and *Trypanosoma*, expulsion of which typically requires a controlled Th1 immunity to prevent the host tissue damage. In fact, the acute immune response to protozoan infection is associated with a strong IFN-γ producing Th1 response (17) associated with the expansion of MDSCs. For instance, in *Trypanosoma cruzi* infection, expansion of MDSCs is regulated by the induction of IFN-γ producing Th1 cells (18). In *Leishmania major* infection, IL12-induced IFN-γ production by Th1 cells promotes resistance to infection and facilitates the expansion of MDSCs (19); MDSCs could also kill the intracellular parasite *L. major* in a NO-dependent manner (20). Although Th1 response plays a critical role against these infections, excessive Th1 response could be detrimental to the host. Thus, MDSCs modulate Th1 inflammatory response in order to protect the host from tissue damage. Consequently, during *T. cruzi* infection, depletion of MDSCs results in an excessive production of IL-6 and IFN-γ, an elevated Th17 response, leading to mortality of the host (21). Similar observations were made in *Toxoplasma gondii* infection, in which an antiparasite Th1 inflammatory response results in extensive intestinal necrosis in the absence of monocytic MDSCs (22).

Host-protective immunity against helminth infections involves a skewed Th2 response associated with elevated levels of MDSCs, as shown during infection with *Schistosoma mansoni* (23, 24), *Taenia crassiceps* (25), *Nippostrongylus brasiliensis* (26), and *Brugia malayi* (27). In fact, MDSCs support a skewed Th2 response by the helminth antigens such as glycans that act as Th2 adjuvants (28). Very recently, it was demonstrated that adoptive transfer of monocytic (CD11b^+^Ly6C^hi^Ly6G^−^) MDSCs, strong suppressors of Th1 responses, failed to protect *N*. brasiliensis-infected mice, whereas granulocytic MDSCs were found to be protective (29). This immunoregulatory role of MDSCs was shown to be mediated by mast cell-derived histamine (30).

## Mregs Directly Induce Host-Protective Th17 Immune Responses

Immunoregulatory functions of Mregs on Th17 differentiation and inflammatory responses have been reported in experimental autoimmune encephalomyelitis (EAE). We showed that the progression of EAE in mice was accompanied by a profound expansion of CD11b^+^Gr-1^+^ MDSCs, which resembled tumor-expanded MDSCs, phenotypically and functionally (31). However, EAE-associated Mregs were found to be highly efficient in producing IL-1β, thereby promoting the differentiation of naive CD4^+^ T cells into Th17 cells. Depletion of Mregs using gemcitabine markedly reduced the severity of EAE as well as Th17 cells and the inflammatory cytokines IL-17A and IL-1β in the lymphoid tissues and spinal cord (31). The pathogenic activities of CCR2^+^Ly6C^hi^ or CD11b^+^Ly6C^hi^ cells, likely due to monocytic Mregs, have also been reported by other studies (32, 33).

Intriguingly, the ability of Mregs to induce Th17 differentiation has also been shown in tumor-bearing mice (34) and patients with ovarian cancer (34). Development of Th17 cells from naive-, memory-, or tumor-infiltrating CD4+ T cells was shown to be driven by Mregs that produce IL-1β/IL-6/IL-23/NO (34). Indeed, recent studies also support a positive correlation between Mregs levels and the levels of Th17 cells or IL-17 production in patients with esophageal cancer (35) or gastrointestinal cancer (36). These new findings not only unmask the different aspects of Mreg functions in the regulation of Th17 cells other than Th1 or Th2 cells, but also highlight the proinflammatory effects of these cells. It is unclear whether the immunosuppressive and proinflammatory activities of Mregs can be uncoupled. However, the proinflammatory feature of these cells may represent a pathogenic factor given the intimate link between inflammation and tumorigenesis, and the progression of inflammatory autoimmune diseases. Although the therapeutic benefits of targeting Mregs in autoimmune disorders remain to be clarified, these studies provide evidence supporting the pleiotropic regulatory effects of Mregs in different contexts.

**Figure 1:**
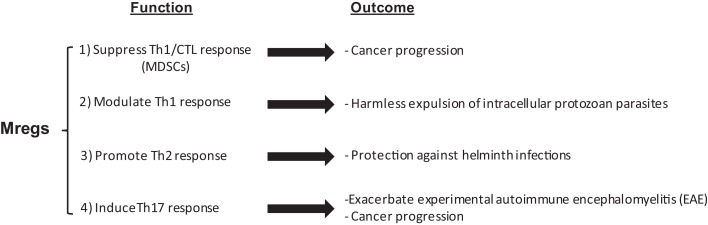
**The multifaceted function of Mregs**.

The multifaceted function of myeloid cells in the exacerbation and amelioration of different diseases associated with the suppression or induction of specific types of the immune response suggests that the term Mregs can better explain their function (Figure 1). In addition, controversial reports on the role of these cells in autoimmune diseases can be consolidated and understood in the context of their regulatory function under certain conditions, which is not merely limited to their immune suppressive function.

## Conflict of Interest Statement

The authors declare that the research was conducted in the absence of any commercial or financial relationships that could be construed as a potential conflict of interest.
